# Data processing software suite *SITENNO* for coherent X-ray diffraction imaging using the X-ray free-electron laser SACLA

**DOI:** 10.1107/S1600577514003439

**Published:** 2014-03-15

**Authors:** Yuki Sekiguchi, Tomotaka Oroguchi, Yuki Takayama, Masayoshi Nakasako

**Affiliations:** a Department of Physics, Faculty of Science and Technology, Keio University, 3-14-1 Hiyoshi, Kohoku-ku, Yokohama 223-8522, Japan; b RIKEN SPring-8 Center, 1-1-1 Kohto, Sayo, Sayo-gun, Hyogo 679-5148, Japan

**Keywords:** coherent X-ray diffraction imaging, X-ray free-electron laser, structure analysis of non-crystalline particles

## Abstract

Coherent X-ray diffraction imaging is a promising technique for visualizing the structures of non-crystalline particles with dimensions of micrometers to sub-micrometers. Recently, X-ray free-electron laser sources have enabled efficient experiments in the ‘diffraction before destruction’ scheme. Diffraction experiments have been conducted at SPring-8 Angstrom Compact free-electron LAser (SACLA) using the custom-made diffraction apparatus KOTOBUKI-1 and two multiport CCD detectors. In the experiments, ten thousands of single-shot diffraction patterns can be collected within several hours. Then, diffraction patterns with significant levels of intensity suitable for structural analysis must be found, direct-beam positions in diffraction patterns determined, diffraction patterns from the two CCD detectors merged, and phase-retrieval calculations for structural analyses performed. A software suite named *SITENNO* has been developed to semi-automatically apply the four-step processing to a huge number of diffraction data. Here, details of the algorithm used in the suite are described and the performance for approximately 9000 diffraction patterns collected from cuboid-shaped copper oxide particles reported. Using the *SITENNO* suite, it is possible to conduct experiments with data processing immediately after the data collection, and to characterize the size distribution and internal structures of the non-crystalline particles.

## Abstract 2

The software suite *SITENNO* is developed for processing diffraction data collected in coherent X-ray diffraction imaging experiments of non-crystalline particles using an X-ray free-electron laser.

## 1. Introduction   

Coherent X-ray diffraction imaging (CXDI) is a technique for visualizing the three-dimensional structures of non-crystalline particles of micrometer to sub-micrometer size (Miao *et al.*, 1998 ▶, 1999 ▶, 2008 ▶). A spatially isolated particle is irradiated by a coherent X-ray beam, and the Fraunhofer diffraction pattern is recorded so that the number of pixels in the detector exceeds more than twice that in the electron density map at the desired resolution, *i.e.* oversampling of the diffraction pattern (Miao *et al.*, 2003*a* ▶). Then, the electron density of the particle is, in principle, obtainable by applying a phase-retrieval algorithm (Fienup, 1982 ▶) to the oversampled diffraction pattern (Miao *et al.*, 1998 ▶, 1999 ▶).

The weak electromagnetic interaction of hard X-rays with electrons in atoms is advantageous for long penetration depths to visualize the structures of thick specimens, and for applying the Born approximation to neglect multiple-scattering effects of X-rays. In addition, because the Ewald sphere of hard X-rays with short wavelengths is approximated as a plane in the small-angle region, the phase-retrieved image corresponds to the electron density of a specimen particle projected along the direction of the incident X-ray beam. Several CXDI experiments demonstrate the potential to visualize structures of non-crystalline particles from material science and biology (Robinson *et al.*, 2001 ▶; Miao *et al.*, 2002 ▶, 2003*b* ▶, 2005 ▶; Song *et al.*, 2008 ▶; Nishino *et al.*, 2009 ▶; Jiang *et al.*, 2010 ▶; Takahashi *et al.*, 2010 ▶).

In 2009, the Linac Coherent Light Source (LCLS), an X-ray free-electron laser (XFEL) source providing X-ray pulses with complete transverse coherence, started user operation (Emma *et al.*, 2010 ▶), and several research groups reported structural analyses of non-crystalline particles, such as large viruses (Seibert *et al.*, 2011 ▶) and soot (Loh *et al.*, 2012 ▶). The extremely intense X-ray pulses with duration of several tens of femtoseconds enable us to record single-shot diffraction patterns of non-crystalline particles at a sufficient signal-to-noise ratio before their destruction at the atomic level (Chapman *et al.*, 2007 ▶; Mancuso *et al.*, 2010 ▶). X-ray pulses come from the LCLS accelerator at a frequency of 120 Hz, a numerous number of diffraction data are automatically acquired in a short period of time, and research groups of LCLS have developed data processing software for XFEL-CXDI experiments (Yoon *et al.*, 2011 ▶; Kassemeyer *et al.*, 2012 ▶; Foucar *et al.*, 2012 ▶; Park *et al.*, 2013 ▶).

**Figure 1:**
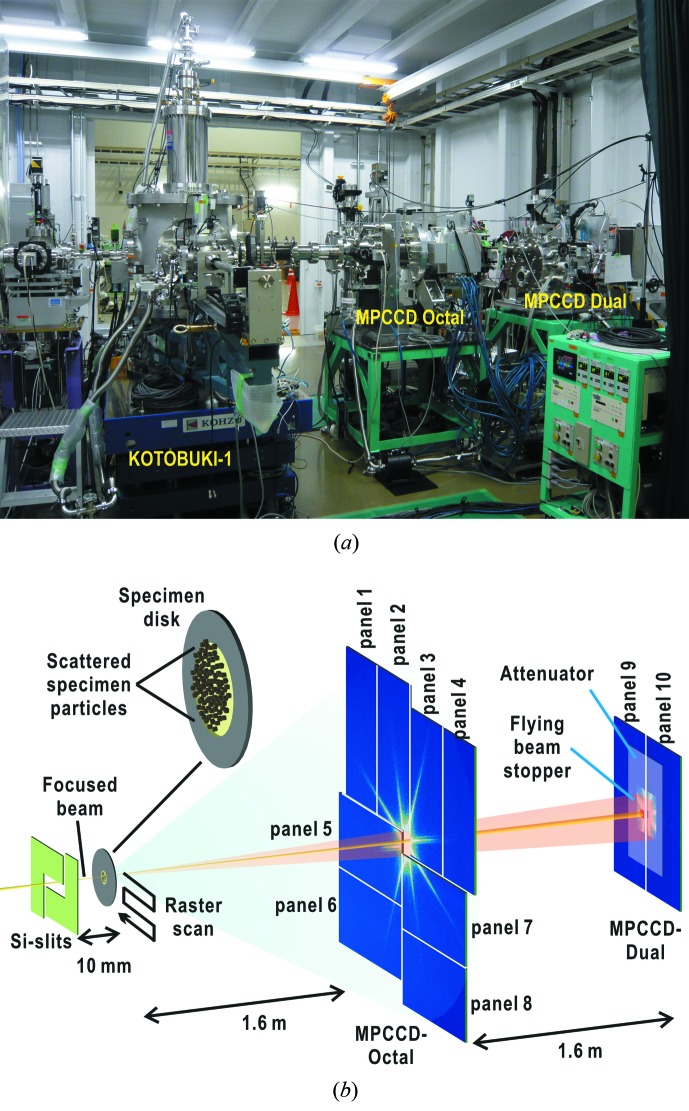
Photograph (*a*) and a schematic illustration (*b*) of our CXDI experiment using the KOTOBUKI-1 apparatus and the MPCCD-Octal and MPCCD-Dual detectors at BL3 of SACLA.

In Japan, SPring-8 Angstrom Compact free-electron LAser (SACLA) facility (Ishikawa *et al.*, 2012 ▶) started user operation in March 2012. Using a diffraction apparatus named KOTOBUKI-1 (Nakasako *et al.*, 2013 ▶) and a pair of multi-port CCD (MPCCD) detectors (Hatsui *et al.*, 2012 ▶), we have been conducting CXDI experiments to collect single-shot diffraction patterns of non-crystalline particles with dimensions of 100–800 nm from material science and biology (Fig. 1*a* ▶). Currently, more than 10000 diffraction patterns are collected within several hours. For structural analysis, raw diffraction data from two detectors have to be merged after corrections with respect to, for instance, the background dark-current noise of the detectors and attenuators used. Manual data processing for such a large amount of diffraction patterns is almost impossible and non-systematic.

We have developed a software suite named *SITENNO* for processing semi-automatically and systematically raw diffraction data and conducting subsequent phase-retrieval calculations. Here we report the details of the algorithms used in the software suite, and the performance of the suite through applying it to a number of diffraction patterns from metal particles obtained in XFEL-CXDI experiments at SACLA.

## 2. Outline of XFEL-CXDI experiments at SACLA   

The KOTOBUKI-1 apparatus is composed of three major components: a vacuum chamber equipped with a goniometer with a cryogenic specimen stage, a loading device to transfer frozen-hydrated specimens to the pot, and an alignment table mounting the chamber and the loading device (Nakasako *et al.*, 2013 ▶). The KOTOBUKI-1 apparatus is placed in experimental hutch 3 of beamline 3 at SACLA so that the specimen position is in the focal spot of a Kirkpatrick–Baez (KB) mirror optics. The mirror optics focuses X-ray pulses to a focal spot with an intensity of approximately 10^10^ to 10^11^ photons µm^−2^ pulse^−1^ (Yumoto *et al.*, 2013 ▶), strong enough to destroy specimen particles at the atomic level (Yumoto *et al.*, 2013 ▶). Specimen particles are randomly scattered on carbon membranes with a thickness of 25–30 nm covering 300 µm-diameter pinholes in molybdenum (or stainless) disks of diameter 3 mm (Okenshoji, Japan) (Takayama & Nakasako, 2012 ▶) or on commercially available Si_3_N_4_ membranes with thicknesses of 50–100 nm (Norcada, Canada; Silson, UK). The goniometer of the KOTOBUKI-1 apparatus can perform raster scans of a specimen disk at a step size of 30–50 µm per pulse to provide particles in the X-ray irradiation area (Fig. 1*b* ▶). During raster-scanning, X-ray pulses stochastically hit particle(s) randomly distributed on a membrane, and the rate of ‘hit’ events depends on the number density of particles.

Single-shot diffraction patterns are recorded simultaneously by two MPCCD detectors in a tandem arrangement (Fig. 1 ▶). One MPCCD sensor is composed of 512 × 1024 pixels of size 50 µm × 50 µm and has eight readout ports. A detector composed of eight MPCCD sensor panels (MPCCD-Octal) is placed at a camera distance of 1.6 m, and collects diffraction patterns in the resolution range of approximately 7–210 nm. The center of the detector is aligned to coincide with the X-ray beam position, and the central aperture of the detector is varied from 3.5 to 9.0 mm by moving four pairs of CCD sensor panels synchronously. Diffracted X-rays in the very small angle region passing through the aperture are recorded by the second detector composed of two MPCCD sensor panels (MPCCD-Dual) located at a camera distance of 3.2 m. A 2 mm × 2 mm beamstop is placed in front of the MPCCD-Dual. This geometry enables us to collect diffraction patterns with a small-angle resolution limit of approximately 480 nm. To extend the dynamic range of the detector system, we use a set of aluminium attenuators of thickness 15–100 µm (corresponding to a transmission of 56–2% for X-rays of photon energy 5.52 keV) between the beam stop and the MPCCD-Dual detector. The central aperture of the MPCCD-Octal and the thickness of the attenuator in front of the MPCCD-Dual can be changed depending on the small-angle diffraction intensities from specimens. The volume of each diffraction pattern is 20 MB [(4 bytes per pixel) × (512 × 1024 pixels) × (10 CCD panels)].

**Figure 2:**
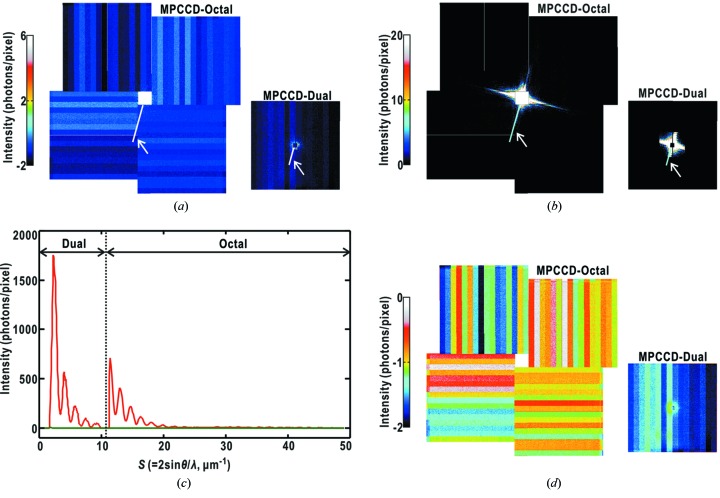
Diffraction patterns of parasite and background scattering recorded without an attenuator (*a*) and from a copper oxide particle with an attenuator of 50 µm thickness (transmission 15%) in front of the MPCCD-Dual detector (*b*). These patterns were collected after positional tuning of the Si slits relative to the incident X-rays. (*c*) Comparison of intensity profiles along the lines indicated by arrows in (*a*) and (*b*). The green and red curves are profiles from (*a*) and (*b*), respectively. (*d*) Thermal and readout noise of detector panels as an average of 100 times accumulation. It should be noted that noise levels are different between readout ports.

A pair of L-shaped silicon frames with beveled edges is equipped approximately 10 mm upstream of the focal point (specimen position) in the KOTOBUKI-1 apparatus (Fig. 1*b* ▶). The frames work as guard corners to eliminate parasite and background scattering from X-ray optics located in the upper stream of the experimental hutch. Through finely aligning the positions of the frames relative to the incident X-rays, parasite and background scattering from the upstream optics are reduced to less than a few tens of photons per pixel around the beamstop even without an attenuator (Fig. 2*a* ▶). The MPCCD-Dual detector usually necessitates an attenuator to collect intense small-angle diffraction patterns. Thus, taking the results shown in Fig. 2(*a*) ▶, background/parasite scattering observed by the MPCCD-Dual detector with an attenuator is negligible in comparison with small-angle diffraction from specimen particles of more than 10^3^ photons per pixel even with attenuators [Figs. 2(*b*) and 2(*c*) ▶]. In this regard, diffraction from a thin membrane support is also negligible as well as the parasite/background scattering. The MPCCD-Octal detector covering the high-angle region is free from parasite scattering and collects weak diffraction patterns. Thus, the thermal and readout noise of the CCD panels, which is independent of diffracted X-ray intensity, is at an intensity level to be taken into consideration in the data processing (Fig. 2*d* ▶).

## 3. Description of the data processing software suite *SITENNO*   

**Figure 3:**
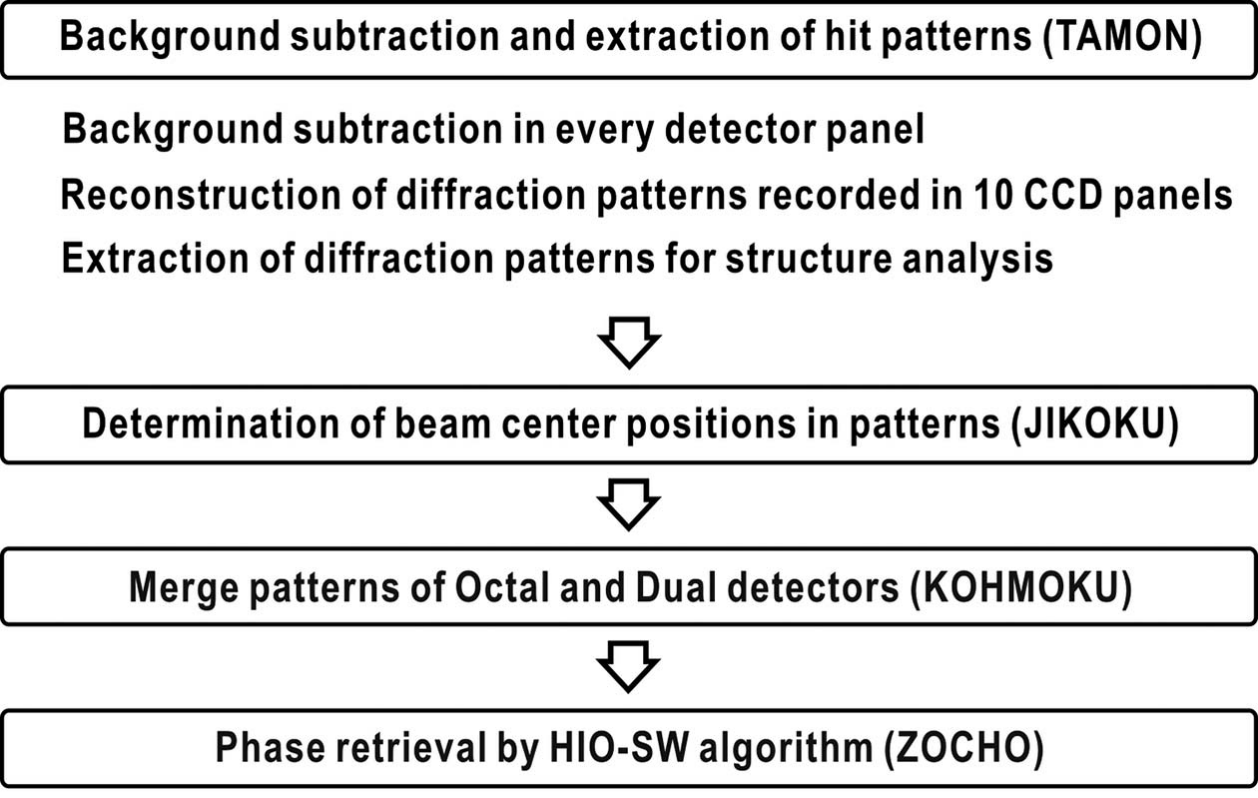
Schematic illustration of the four processing steps for raw diffraction data by the *TAMON*, *JIKOKU*, *KOHMOKU* and *ZOCHO* subprograms of the *SITENNO* suite for a few thousands of diffraction patterns collected through a raster scan of the specimen disk (Fig. 1*b* ▶).

The *SITENNO* software suite written using the FORTRAN90 language is composed of three subprograms, named *TAMON*, *JIKOKU* and *KOHMOKU*, to process raw diffraction patterns to data files suitable for phase-retrieval calculations by another subprogram named *ZOCHO* (Fig. 3 ▶). We use the *ImageJ* program (Schneider *et al.*, 2012 ▶) to view the diffraction patterns and phase-retrieved projection images. In the following sections, we describe the details of the algorithms used in the subprograms.

### 3.1. Background subtraction, reconstruction and extraction of diffraction patterns   

The subprogram *TAMON* processes raw diffraction data through three steps: reconstruction of diffraction patterns from data from ten CCD panels, background subtraction, and extraction of diffraction patterns suitable for the subsequent processing and analysis (Fig. 3 ▶).

In every raster scan, diffraction patterns from the data acquisition system of the MPCCD detectors are converted to a single file in HDF5 format (http://www.hdfgroup.org/HDF5), which contains all diffraction patterns by X-ray pulses provided during the scan. First, *TAMON* reconstructs image data from the eight panels of the MPCCD-Octal detector and the two from the MPCCD-Dual into a single file using the geometrical parameters describing the relative positions and orientations of the detector panels (Kameshima, 2012 ▶). The positional and angular accuracies of parameters are approximately 25 µm and 1 mrad, respectively. In addition, the diffraction intensity given in analog-to-digital units is converted to photons. The geometrical parameters and the conversion constant depending on X-ray energy are available from the detector group of SACLA.

Diffraction patterns are almost free from parasite, background and membrane support scattering as described in the previous section (Fig. 2 ▶). Thus, we are concerned with two types of background noise in the MPCCD detectors. One is thermal and readout noise (Fig. 2*d* ▶). Before starting every raster scan for data collection, we record 100 noise patterns without X-ray beam using the two MPCCD detectors operated under the same operation conditions as with data collection. The patterns are averaged and subtracted from all diffraction patterns obtained in the raster scan.

**Figure 4:**
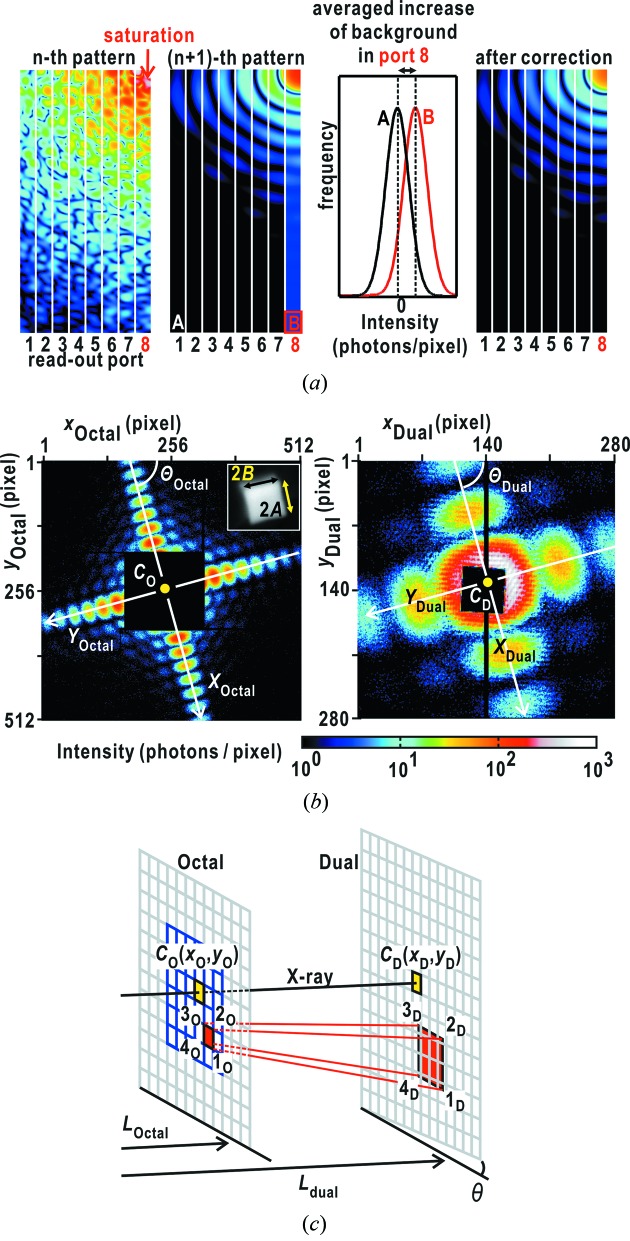
Schematic illustration of some of the algorithms used in the *SITENNO* suite. (*a*) Schematic illustration regarding background subtraction for a detector readout port (port 8 in this illustration) containing pixels receiving X-ray photons exceeding the saturation limit. For instance, the saturation limit for X-rays of energy 5.5 keV is approximately 2500 photons per pixel. Just after some pixels in a readout port (here, for instance, port 8) are in saturation in the *n*th diffraction pattern (left panel), the noise of all pixels in the port increases by approximately one photon in subsequently collected patterns (the second panel from the left). The average increase of the noise in the port is evaluated by comparing the frequency distribution (red line in the graph) of readout counts in ROIs of 64 × 50 pixels placed at the edges of the port (B in port 8) with that (black line) in a port free from saturation events (A in port 1). We obtain the diffraction pattern shown in the right-hand panel by subtracting the average value from the raw data. (*b*) The orthogonal coordinates, *X*_Octal_ and *Y*_Octal_ (*X*_Dual_ and *Y*_Dual_), used to approximate the Fraunhofer diffraction pattern from a cuboid-shaped copper oxide particle with edge lengths of 2*A* and 2*B* as shown in the inset of the left-hand panel. Parameter Θ_Octal_ (Θ_Dual_) represents the angle between the coordinate and the MPCCD-Octal (MPCCD-Dual) detector edge. (*c*) Schematic illustration to calculate how many pixels on the MPCCD-Dual detector correspond to one pixel on the MPCCD-Octal detector. The parameters and calculation details are described in the text.

The other background noise appears when CCD pixels are saturated by the incidence of intense X-rays (Fig. 4*a* ▶). Then, noise increases by approximately one photon over the readout ports, which contain the saturated pixels. The increase is unfortunately kept during the subsequent data collection, until the readout port is electrically reset. The increase in noise is small in each pixel, but the accumulated value over thousands of pixels becomes large. Because we use the sum of the diffraction intensity of regions of interest (ROIs) in the small-angle region to judge whether diffraction patterns are worth being analyzed, the accumulated value may mislead us to erroneous judgment that diffraction patterns smeared by the background noise are suitable for structural analyses despite weak and unclear speckle patterns.

When *TAMON* identifies a readout port with saturated pixels, it automatically creates a frequency distribution regarding background intensity over approximately 3000 pixels in an edge region located at high diffraction angle in the port. Because the edge region is almost free from photons diffracted by specimen particles, the distribution has a peak at zero intensity without an increase in readout noise. In contrast, an increase in readout noise occurs, as indicated by the peak shift of the distribution by one photon (Fig. 4*a* ▶). Thus, *TAMON* subtracts the value from intensity data over the pixels in the readout port in every diffraction pattern recorded after the saturation event.

In the next step, the *TAMON* subprogram sorts the background-subtracted diffraction patterns with respect to the sum of small-angle diffraction intensities in ROIs defined by the user. We determine the threshold value to extract diffraction patterns for the subsequent processing through graphically examining the sorted diffraction patterns with respect to the signal-to-noise ratio and the visibility of speckle patterns up to a resolution desired for structure analyses. Then, *TAMON* extracts diffraction patterns with the summed intensity values exceeding the threshold given by the user. The threshold value determined for a specimen is applicable to the other specimens prepared from the same source.

### 3.2. Direct-beam positions in diffraction patterns   

The direct-beam positions are indispensable for merging diffraction patterns from the two MPCCD detectors. The major role of the *JIKOKU* subprogram is the determination of direct-beam positions in diffraction patterns recorded by the two MPCCD detectors. To find direct-beam positions in diffraction patterns, *JIKOKU* uses two different methods.

The first method assumes that the direct-beam position is stable for a long period of time. Direct-beam positions of diffraction patterns are determined using the diffraction patterns from cuboid-shaped nanoparticles. When a cuboid-shaped particle adsorbs onto a membrane so that one of the six faces is normal to the incident X-rays, the diffraction pattern is approximated by the Fraunhofer diffraction from a rectangular-shaped aperture (Born & Wolf, 1999 ▶). The diffraction intensity *I* at scattering vector 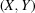 from an aperture with edge lengths of *2A* and *2B* is given by the following equation,
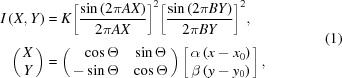
where 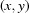 is the position of a detector pixel, and 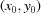 is the direct-beam position. The origin-shifted pixel position 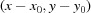 is converted to the scattering vector 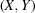 using parameters 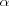 and 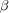 calculated from the camera length, and the rotation matrix with respect to the angle 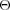 between the detector edge and the *X* axis (Fig. 4*b* ▶). Parameter *K* is an intensity scale factor between the observed and calculated diffraction patterns.

At the beginning of XFEL-CXDI experiments, we collect diffraction patterns from cuboid-shaped copper oxide particles of dimensions 100–600 nm (Kuo *et al.*, 2007 ▶) and select the best with respect to the intensities and the visibility of the speckle patterns. Using the *SALS* subroutine for non-linear least-squares calculation (Nakagawa & Oyanagi, 1980 ▶) incorporated into the *JIKOKU* subprogram, we first determine a set of parameters (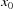, 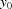, 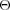, *A*, *B* and *K*) for the best diffraction pattern recorded by the MPCCD-Dual detector. Using the edge length of the particle, we next determine another set of parameters (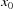, 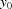, 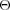 and *K*) for the diffraction pattern simultaneously recorded by the MPCCD-Octal detector. An initially determined direct-beam position will be applied to diffraction patterns collected until the occurrence of drifts of the direct-beam position exceeding more than one pixel (50 µm) at the MPCCD-Octal detector.

The second method for estimating direct-beam positions is based on the Friedel symmetry of diffraction patterns in the small-angle region, where the Ewald sphere of used X-rays is approximated as a plane. Small-angle diffraction patterns from specimen particles free from anomalous effects are ideally identical between symmetry-related ROIs. Here we measure quantitatively the similarity of diffraction patterns between symmetry-related ROIs using the following score (Fujioka *et al.*, 2008 ▶),
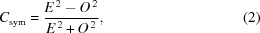
with 
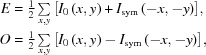
where 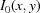 is the intensity in a targeted ROI and 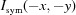 is that in the symmetry mate with respect to a pixel assumed to be the center-of-symmetry of the targeted diffraction pattern. When a pixel assumed as the center-of-symmetry in applying equation (2) coincides with the direct-beam position in the pattern, the *C*_sym_ score reaches the highest value (ideally equal to 1). Thus, this method is applicable for searching direct-beam positions of any diffraction patterns, even when the X-ray beam fluctuates pulse by pulse. However, because the ambiguity of the *C*_sym_ score is inversely proportional to the diffraction intensity of targeted ROIs, reliable direct-beam positions will be obtainable from diffraction patterns composed of clear and intense speckles.

After determining the direct-beam position, the *JIKOKU* subprogram can generate diffraction patterns of a symmetry-related area. For instance, we can apply this function to evaluate how the diffraction pattern satisfies the Friedel symmetry, and to generate diffraction patterns in detector areas lacking diffraction patterns, such as the gaps between detector panels or overlaps in edges of detector panels. Sometimes, a few narrow areas of, for instance, approximately 3 × 150 pixels still lack diffraction patterns even after applying the symmetry operation [see the border between the Dual and Octal MPCCD detectors in Fig. 7(*a*)]. We exclude those areas in the subsequent phase-retrieval calculations because we confirmed little influence of the absence to phase-retrieved density maps through simulation studies.

### 3.3. Merge of diffraction patterns from two MPCCD detectors   

The *KOHMOKU* subprogram merges the diffraction patterns of the two detectors. First, the diffraction patterns of the two MPCCD detectors are superimposed with respect to the beam centers. Then, *KOHMOKU* calculates how many pixels in the diffraction pattern of the MPCCD-Dual detector correspond to one pixel of the MPCCD-Octal (Fig. 4*c* ▶). For the first calculation, each corner position of a pixel assumed in the aperture of the MPCCD-Octal detector coordinate is converted to the coordinate of the MPCCD-Dual detector,
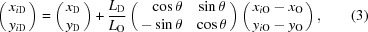
where 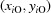 and 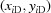 are the positions of the *i*th corner in the pixel coordinate system of the MPCCD-Octal and the MPCCD-Dual detectors, respectively. 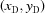 is the direct-beam position in the MPCCD-Dual detector, and 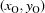 is that in the MPCCD-Octal detector. 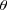 is the rotation angle of the MPCCD-Dual detector relative to that of the MPCCD-Octal detector, and is the difference between the 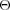 parameters of the two MPCCD detectors in equation (1). 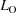 and 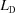 are the camera lengths of the MPCCD-Octal and the MPCCD-Dual detectors, respectively.

The diffraction intensity contained in the square, *i.e.* the diffraction intensity that would be observed in the pixel of the MPCCD-Octal detector, is summed over the area covered by the square. When only part of the pixel is covered by the square, the intensity is calculated by weighting the areal ratio of the part to the pixel. Finally, the intensity scale factor, which is calculated from the thickness of an attenuator used in front of the MPCCD-Dual detector, is applied to the diffraction pattern converted from that of the MPCCD-Dual detector.

### 3.4. Phase-retrieval calculation   

The *ZOCHO* subprogram for phase-retrieval calculation has been developed through simulation studies on structure analyses of macromolecules in aqueous environment from single-shot diffraction data (Kodama & Nakasako, 2011 ▶; Oroguchi & Nakasako, 2013 ▶). This subprogram implements the hybrid input–output (HIO) algorithm (Fienup, 1982 ▶) in combination with the shrink-wrap (SW) algorithm (Marchesini *et al.*, 2003 ▶).

In this study, we performed phase-retrieval calculations only once for any merged diffraction pattern. Prior to the calculation, the diffraction pattern is trimmed to a 256 × 256 pixels size, and reduced further to a size of 128 × 128 through a 2 × 2 binning to increase the signal-to-noise ratio of diffraction. Although the oversampling ratio reduced to a quarter of the original, the noise reduction was effective for success in the phase-retrieval calculation for most of the diffraction patterns treated here. It should be noted that, when a binning area contains at least one pixel lacking diffraction intensity, the diffraction intensity of the binned area is set to be zero and excluded in the subsequent phase-retrieval calculation.

The resolution at the edge of the processed diffraction pattern is 57.4 nm. The initial support is given by the auto-correlation function of the diffraction pattern. In the phase-retrieval calculation, the support update by SW algorithm was conducted for every 100 HIO cycles, and this procedure was iterated 100 times. Furthermore, we apply an additional HIO calculation of 1000 cycles to the electron density obtained by the HIO–SW calculation. The progress of the phase-retrieval calculation and the quality of the retrieved projection electron density map are monitored by the parameter γ (Miao *et al.*, 2001 ▶, 2003*a* ▶), which is the ratio between the total electron densities inside and outside of the support, as defined by 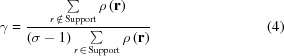
where 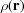 is the electron density and σ is the oversampling ratio. In addition, we compared the diffraction amplitudes calculated from a phase-retrieved electron density map with the observed amplitudes using
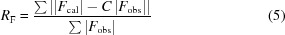
where 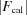 and 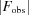 represent the Fourier modulus of the phase-retrieved density map and experimentally observed diffraction amplitude, respectively, and *C* is a scale factor. The sum is taken up to the resolution limit of the diffraction pattern analyzed.

### 3.5. Computation and data output   

Data processing and phase-retrieval calculations are carried out immediately after data collection using the *SITENNO* suite, which is currently installed on a supercomputer system, named SACLA-HPC, composed of 960 cores of Intel Xeon CPU X5690 (3.47 GHz per core). Among the four subprograms, the *ZOCHO* subprogram has been parallelized by TO for efficient and fast calculation by supercomputer, particularly in the calculation of the two-dimensional fast Fourier transformation (2D-FFT) as follows.

To reduce the memory access and the computational costs during 2D-FFT for a density map of 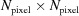 [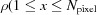, 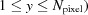] to the structure factor [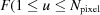, 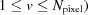], a 2D-FFT program is developed to efficiently utilize several computational cores (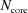). The map is divided into 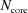 stripes, and the *i*th stripe, 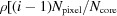, 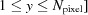, is processed on the *i*th core. A function from one-dimensional FFT (1D-FFT) regarding *y* for the stripe on the *i*th core is divided further into 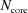 blocks. The function in the *j*th block on the *i*th core is expressed as 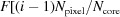 < *x* ≤ 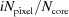, 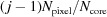 < *v* ≤ 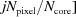. When the *j*th block on the *i*th core is exchanged with the *i*th block on the *j*th core through the message passing interface of the computer system, the function appearing in the *i*th core becomes 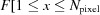, 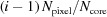 < *v* ≤ 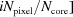. Through the 1D-FFT regarding *x* in the *i*th core, we obtain the structure factor of 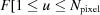, 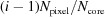 < *v* ≤ 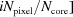. This calculation process is also applied to the inverse 2D-FFT of structure factors to density maps. Phase-retrieval calculation utilizing this 2D-FFT method on 12 cores is approximately ten times faster than that with ordinary 2D-FFT on one core.

In SACLA-HPC, each node is composed of 12 cores, and the scalability of the phase-retrieval calculation speed by the *ZOCHO* subprogram is almost linear for a diffraction pattern of 128 × 128 pixels. Therefore we used 12 cores for the phase-retrieval calculation of each diffraction data. In addition, for maximum extent in the on-the-fly analysis of large experimental data, the *ZOCHO* subprogram automatically submits phase-retrieval jobs to idling nodes through monitoring the status of nodes in the SACLA-HPC.

**Figure 5:**
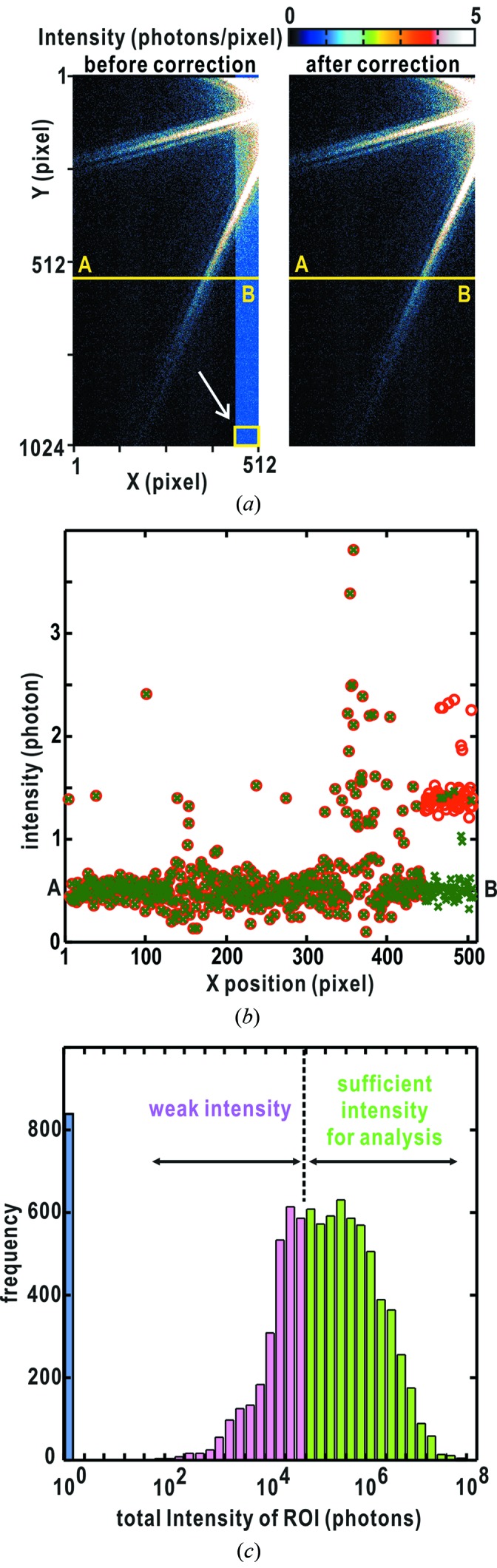
Results from the data processing by the *TAMON* subprogram. (*a*) Diffraction patterns before (left) and after (right) background correction in the readout port, in which some pixels received X-ray photons exceeding the saturation limit. (*b*) Line profiles before (red circles) and after (green crosses) the correction between points A and B of the diffraction patterns in (*a*). (*c*) Histogram of the diffraction intensities of a user-defined ROI for 8738 diffraction patterns from cuboid-shaped copper oxide samples. Blue, pink and green histogram bars indicate no signal (820 diffraction patterns), below (3389) and above (4529) the given threshold intensity value, respectively.

**Figure 6:**
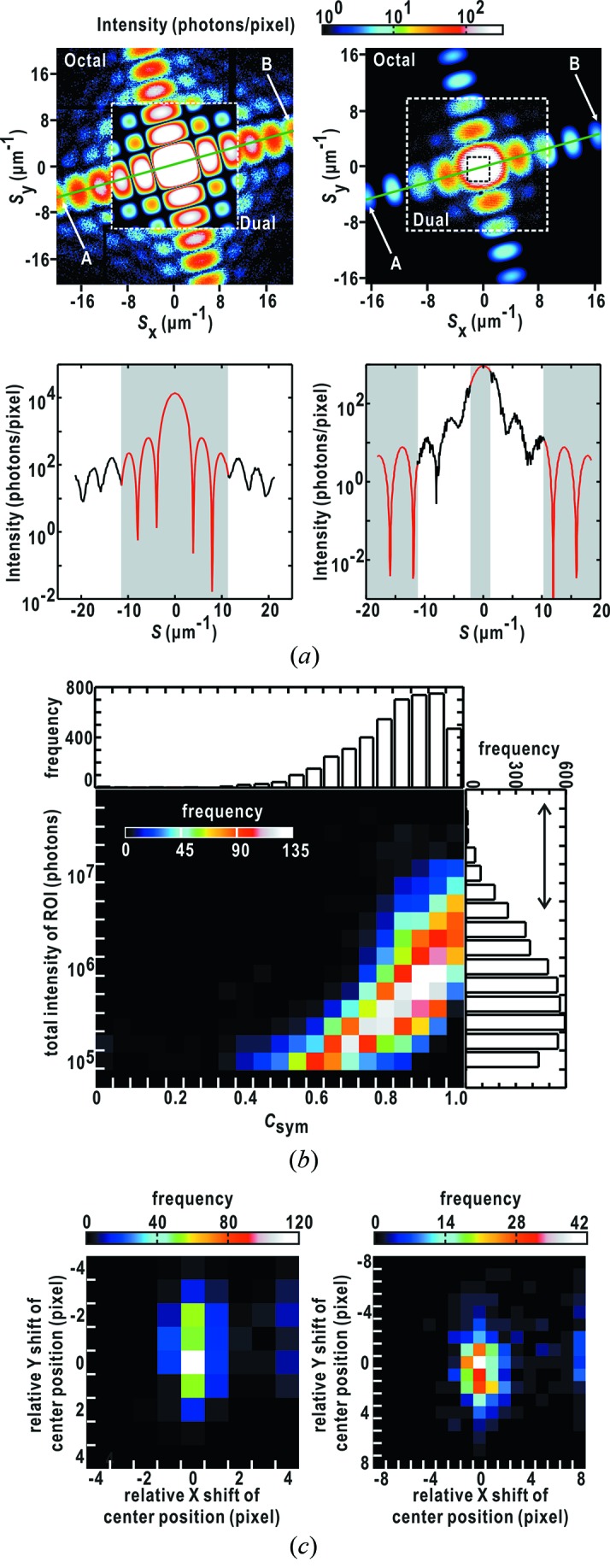
Results from the data processing by the *JIKOKU* subprogram. (*a*) Determination of parameters in equation (1) by fitting the diffraction pattern of a cuboid-shaped copper oxide particle simultaneously recorded in the MPCCD-Octal (outside the white dotted box in the left-hand panel) and the MPCCD-Dual (inside the white dotted box in the right-hand panel) detectors. The experimental diffraction patterns are shown with those calculated from the determined values of the parameters (Table 2 ▶). The line profiles along positions A and B in the upper panels are shown in semi-log plots to emphasize the continuity in the borders between the experimental and theoretical data. The regions of theoretical prediction are indicated by the gray-colored background. (*b*) Distribution of 4529 diffraction patterns classified with respect to the diffraction intensity and *C*_sym_ scores. (*c*) Distribution of pixel positions assigned as center-of-symmetry searched using *C*_sym_ scores for diffraction patterns with the 500 highest intensities (indicated by an arrow) in (*b*) in the MPCCD-Octal detector (left) and the MPCCD-Dual detector. The search is carried out for pixels around the direct-beam position (0, 0) determined by equation (1).

**Figure 7:**
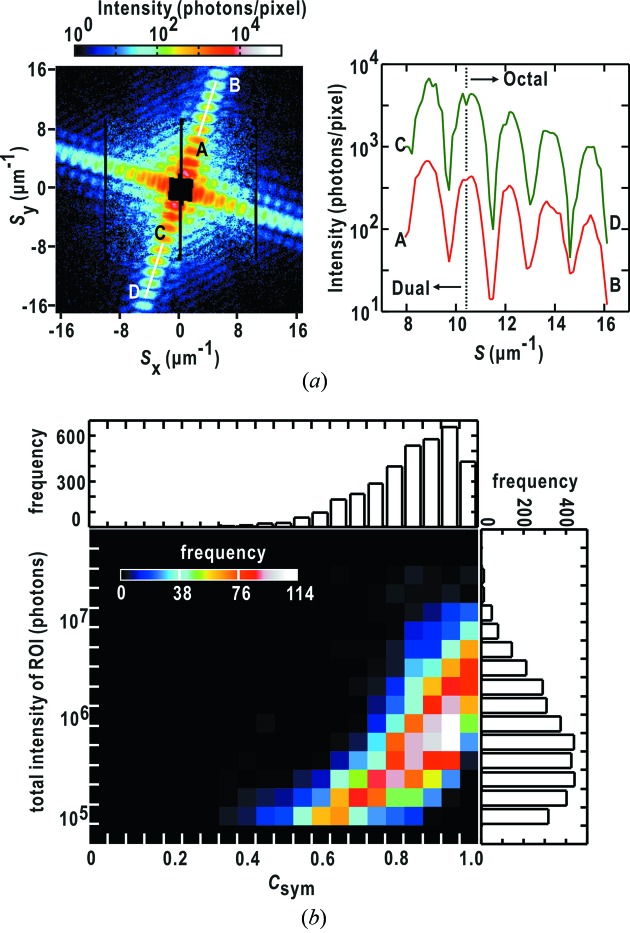
Results from data processing by the *KOHMOKU* subprogram. (*a*) Merged diffraction pattern from a copper oxide particle (left). The line profiles along A–B and C–D are illustrated in the right-hand panel. The profiles of C–D are shifted appropriately for clarity. (*b*) Classification of 3508 successfully merged diffraction patterns with respect to the *C*_sym_ scores and the diffraction intensity in the small-angle region.

**Figure 8:**
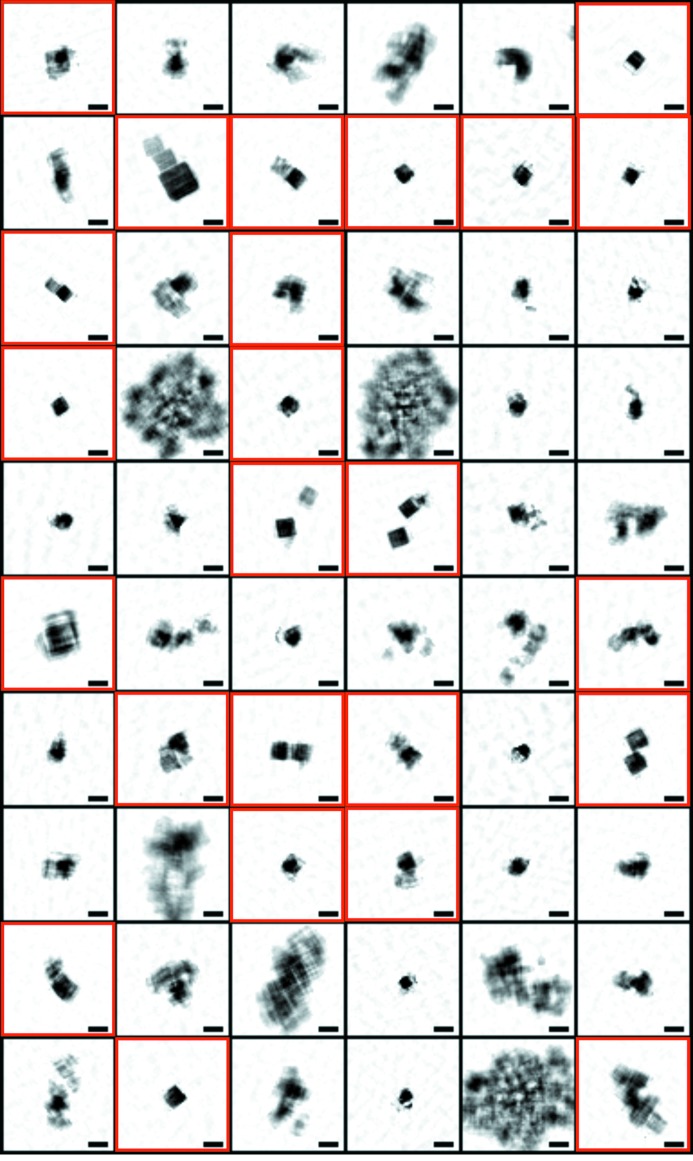
Results from the phase-retrieval analyses by the *ZOCHO* subprogram: a set of 60 electron density projection maps obtained by applying the *ZOCHO* subprogram. The scale bar indicates 300 nm. The red boxes indicate successfully retrieved electron density maps.

The three subprograms for raw data processing output several files written in the ‘csv’ format suitable for monitoring the progress of the data processing as shown in Figs. 5–8. The *ZOCHO* subprogram outputs electron density maps and diffraction patterns as binary image data. These image files are visualized easily using the *ImageJ* program.

## 4. Diffraction data collection   

### 4.1. Sample preparation   

Copper oxide particles were prepared according to the literature (Kuo *et al.*, 2007 ▶). We first made a mixture of 1 m*M* copper sulfate and 33 m*M* sodium dodecyl sulfate solutions. Sodium ascorbate solution of 200 m*M* was added to the mixture and stirred for 5 s. Finally, 1 *M* sodium hydroxide solution was mixed to the solution and stirred again for 5 s. We iterated this procedure several times to produce copper oxide particles of 100–600 nm. Particles were scattered and dried on Si_3_N_4_ membrane with a 3 mm × 3 mm window at a number density of approximately 5 particles per 10 µm × 10 µm. Prior to CXDI experiments, the size distribution of the prepared particles was analyzed for 100 images taken using a TM3000 scanning electron microscope (SEM) (Hitachi High-Tech, Japan).

### 4.2. XFEL-CXDI experiment for copper oxide particles at SACLA   

The diffraction patterns of cuboid-shaped copper oxide particles were collected using X-rays with a photon energy of 5.52 keV focused to a spot size of approximately 1.9 µm × 1.9 µm full width at half-maximum. The repetition rate of XFEL pulses was reduced from 10 Hz to 1 Hz by a pulse selector. Each Si_3_N_4_ membrane adsorbing copper oxide particles was mounted on a goniometer in the vacuum chamber of the KOTOBUKI-1 apparatus. Raster scanning of the membrane relative to the focused XFEL pulses was conducted at a step of approximately 50 µm interval per pulse (Nakasako *et al.*, 2013 ▶). We set an aluminium attenuator of 50 µm thickness (transmittance 14.8% for 5.52 keV X-rays) in front of the MPCCD-Dual detector.

## 5. Results   

Here we describe the performance of each subprogram in the *SITENNO* suite through processing a set of 8738 single-shot diffraction patterns of cuboid-shaped copper oxide particles from 14 specimen membranes. The CPU times necessary for data processing by the four subprograms are shown in Table 1 ▶.

### 5.1. Background subtraction and extraction of diffraction patterns   

During the collection of 8738 single-shot diffraction patterns, saturation events occurred 204 times in the MPCCD-Octal detector and 1531 times in the MPCCD-Dual detector even with a 50 µm attenuator. As the representative example shown in Figs. 5(*a*) and 5(*b*) ▶, 8122 diffraction patterns collected after the saturation events were processed to eliminate the increased background in readout ports according to the scheme shown in Fig. 4(*a*) ▶. The diffraction intensity depended on the size and the number of copper oxide particles in the irradiation area as well as the intensity of the focused X-rays varying pulse by pulse. Clusters of copper oxide particles particularly tended to induce saturation of pixels in the MPCCD-Dual detector collecting small-angle diffraction patterns, because of their large total scattering cross section.

Background-subtracted diffraction patterns were sorted with respect to the diffraction intensity summed in a ROI of 512 × 512 pixels centered at the direct-beam position. At this stage, we tentatively assumed that the direct-beam position coincided with the center of the MPCCD-Octal detector. The square-shaped ROI covers a resolution of approximately 88–29 nm. While 7918 X-ray pulses hit single or clusters of copper oxide particles, particles were absent from the irradiation area for 820 pulses. For a given threshold of approximately 7 × 10^4^ photons, *TAMON* extracted 4529 diffraction patterns for the subsequent data processing (Fig. 5*c* ▶).

### 5.2. Direct-beam positions in diffraction patterns   

Direct-beam positions were first determined by applying equation (1) to the diffraction pattern of a cuboid-shaped copper oxide particle with approximate dimensions of 250 nm [Fig. 6(*a*) ▶ and Table 2 ▶]. The positions of diffraction maxima, which were calculated using the determined particle size and using the direct-beam position, were consistent with those observed (Fig. 6*a* ▶). The in-plane rotation of the MPCCD-Dual detector relative to the MPCCD-Octal detector was 0.37°.

Next we examined the stability of the direct-beam position using equation (2). Prior to the examination, we found the correlation that diffraction patterns composed of clear and intense speckle peaks give theoretically ideal *C*_sym_ scores reflecting the Friedel symmetry through analyzing the extracted 4529 diffraction patterns (Fig. 6*b* ▶). Thus, for unambiguously searching a pixel located at the center-of-symmetry in a diffraction pattern, ideally coinciding with the direct-beam position, we selected diffraction patterns with the 500 highest intensities among the extracted patterns. As a result, pixels assumed as center-of-symmetry giving the highest *C*_sym_ score in each diffraction pattern distributed within 1–2 pixels around the direct-beam positions in the MPCCD-Octal detector and within 2–4 pixels in the MPCCD-Dual detector (Fig. 6*c* ▶). Finally, the direct-beam positions of the 4529 diffraction patterns were examined for a 3 × 3 pixel area in the diffraction patterns from the MPCCD-Octal detector, and for a 5 × 5 pixel area in the patterns from the MPCCD-Dual detector. Because 2 × 2 pixels in the MPCCD-Dual detector correspond to approximately one pixel in the MPCCD-Octal detector in our experimental set-up, the apparent direct-beam position estimated using *C*_sym_ scores is stable within approximately 50 µm × 50 µm at the MPCCD-Octal position.

### 5.3. Merged diffraction patterns from two MPCCD detectors   

Fig. 7(*a*) ▶ shows a diffraction pattern of a single copper oxide particle reconstructed from those recorded by the two MPCCD detectors. The intensity line profiles connected smoothly between the diffraction patterns recorded by the two detectors. Even at the borders around gaps between detector panels, the diffraction intensity varied as expected.

In the present study, we monitored the smoothness of the profiles to judge whether diffraction patterns from the two MPCCD detectors were merged well. Among 4529 diffraction patterns extracted by the *TAMON* subprogram, 3508 diffraction patterns were judged as successfully merged taking the smoothness of the profiles at the border regions of the two different detectors. This processing depends on whether the direct-beam positions were correctly determined. Thus, as demonstrated in Fig. 7(*b*) ▶, most of the successfully merged diffraction patterns with clear and intense speckle patterns tended to give the sum of intensity beyond 5 × 10^5^ photons in a specified ROI and *C*_sym_ scores larger than 0.8.

### 5.4. Phase-retrieval calculation   

Fig. 8 ▶ shows a representative set of 60 phase-retrieved electron density projection maps obtained by HIO–SW calculation. Electron density maps are classified into two categories. Maps in the first category contain electron densities of rectangular or square shapes assignable as copper oxide particles. In the other category, support of electron density expands to larger than the size expected from the speckle size and out of shape. As a result, out of 3508 successfully merged diffraction patterns, *ZOCHO* retrieved 1276 electron density projection maps of copper oxide particles with an averaged γ value of 0.0387 and *R*_F_ of 0.2238.

**Figure 9:**
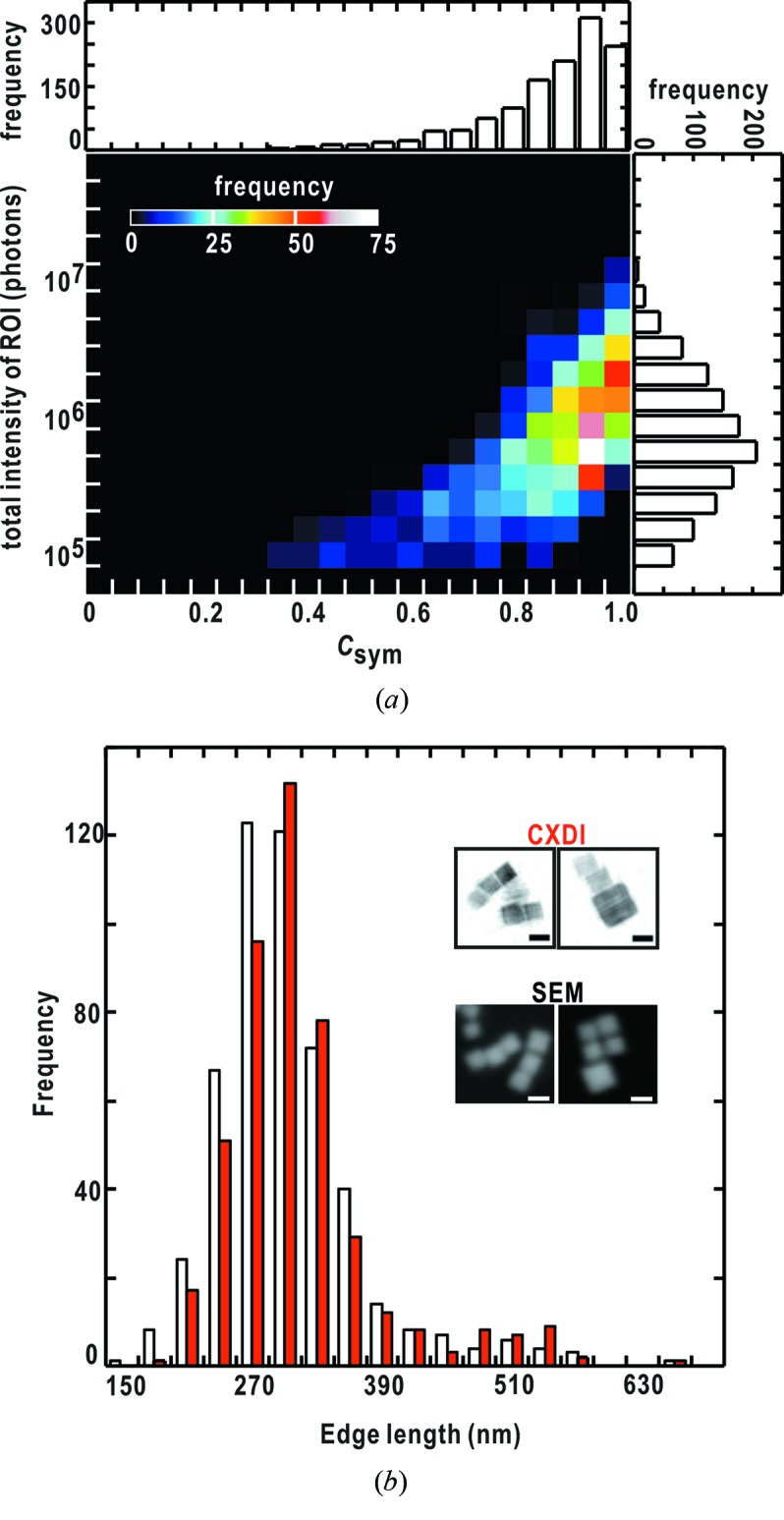
(*a*) Distribution of the number of diffraction patterns, the electron density maps from which are successfully retrieved by *ZOCHO*, plotted with respect to the *C*_sym_ scores and diffraction intensity in the small-angle region. (*b*) Comparison of the size distribution of copper oxide particles in SEM and CXDI (white and red colored histogram bars, respectively). The inset shows assemblies of copper oxide particles in SEM and phase-retrieved electron density maps (labeled as CXDI) from diffraction patterns. The scale bar indicates 300 nm.

In most of the diffraction patterns, the electron densities of which were retrieved, the *C*_sym_ scores were better than 0.8 and diffraction intensities in the specified ROIs in the small-angle region were larger than 5 × 10^5^ photons (Fig. 9*a* ▶). Diffraction patterns that failed to be phase-retrieved are roughly classified into three groups. Diffraction patterns in the first group are composed of weak speckle peaks. In the second group, many pixels in the small-angle region around the beamstop lose diffraction intensities because the diffraction intensities exceed the saturation limit. In the third group, diffraction patterns are composed of very fine speckle patterns probably originating from several particles separated by more than 1 µm. It is very difficult to retrieve electron density maps because of the small oversampling ratio that is insufficient to apply the HIO–SW calculation.

### 5.5. Size distribution of copper oxide particles   

As one of applications of the *SITENNO* suite to explore structural analyses using XFEL-CXDI experiments, we here try to illustrate the size distribution of copper oxide particles. The phase-retrieved 1276 electron density maps (Fig. 8 ▶) indicated that the copper oxide particles synthesized under the solution condition were varied in dimensions. The maximum edge lengths of particles are directly measured as the number of pixels in the phase-retrieved electron density maps, the size of which is half of the resolution in the phase-retrieval calculation.

The size distribution of copper oxide particles was constructed as shown in Fig. 9(*b*) ▶. The distribution has a major peak at 300 nm with a half-width of 60 nm and an additional small peak at 510 nm. The profile of this distribution from the CXDI experiments is similar to that observed in SEM images of specimen particles synthesized under the same conditions. The major and minor maxima are consistent between the two methods, except for the difference in the number of particles between 210 and 270 nm.

## 6. Discussion   

The *SITENNO* suite (Fig. 3 ▶) has been developed to process a large number of single-shot diffraction data collected using the KOTOBUKI-1 apparatus and MPCCD detectors at SACLA (Fig. 1 ▶), and the performance of the suite is examined through the structure analysis of copper oxide particles with dimensions of 100–600 nm (Figs. 5–9).

The three subprograms for raw data processing work well and process 1000 diffraction patterns within 26 min. In the near future, we must improve the performance of the diffraction apparatus to accommodate the pulse repetition rate of 30 Hz currently available at SACLA. The performance of the *SITENNO* suite presented in this study would guarantee that the suite can accommodate the huge amount of diffraction data through parallel processing on the SACLA-HPC. In addition, the present analysis suggests that the algorithm used in the current version of *SITENNO* requires diffraction patterns with good signal-to-noise ratio for finding direct-beam positions (Fig. 6 ▶), merging diffraction patterns of two CCD detectors (Fig. 7 ▶) and obtaining correct electron densities of particles (Figs. 8 ▶ and 9 ▶).

Here we discuss the benefits, feasibility, limitations and future improvements of the *SITENNO* suite toward performing XFEL-CXDI experiments efficiently.

### 6.1. Determination of the direct-beam position   

A wide dynamic range of the detector system to record small-angle diffraction patterns requires the use of two MPCCD detectors operated under different attenuation conditions and the development of software to merge the diffraction patterns. To merge the diffraction patterns, the direct-beam positions in the diffraction patterns are required with a precision of better than the CCD pixel size. In the present study, we first determine the direct-beam positions in diffraction patterns using equation (1) and verify them using equation (2) (Fig. 6 ▶). The ambiguity in beam center positions estimated using equation (2) depends on the clarity and intensities of speckle patterns composing diffraction patterns. Anisotropic diffraction patterns such as those observed for cuboid-shaped particles (Figs. 6 ▶ and 7 ▶) are sometimes out of the ROIs placed for counting diffraction intensities. This causes an ambiguity in determining the center-of-symmetry diffraction patterns of copper oxide cubes.

**Figure 10:**
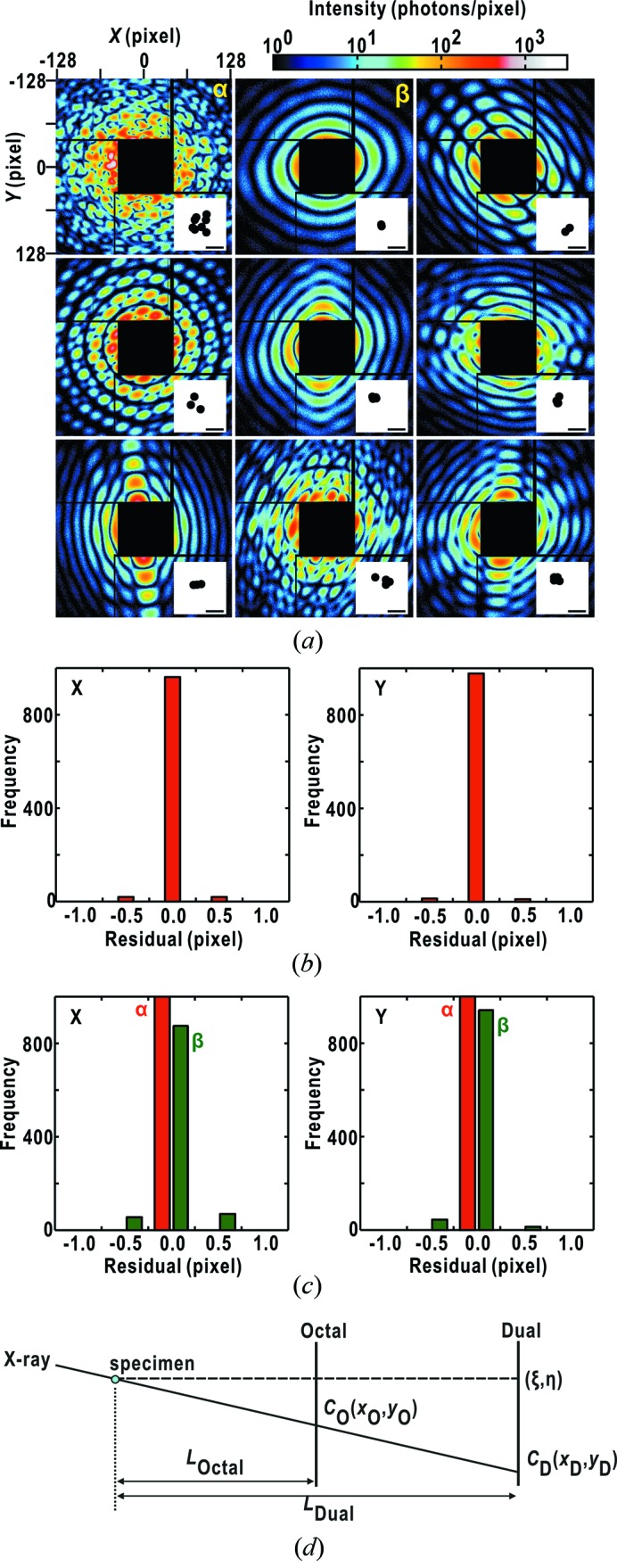
Simulation examining the accuracy in estimating the direct-beam positions in diffraction patterns recorded by the MPCCD-Octal detector using equation (2). Diffraction patterns were calculated for 1000 models generated by randomly placing one or several circles of diameter 140 nm. Each diffraction pattern is converted to the pixel coordinate of the Octal detector by randomly shifting the direct-beam position within ±10 pixels. After scaling the maximum diffraction intensity among the 1000 diffraction patterns to the maximum photon number acceptable in the MPCCD pixel (2500 X-ray photons), the diffraction pattern was smeared by Poisson noise. (*a*) Representative nine diffraction patterns shown with the electron density models in the inset on the lower right. The scale bar corresponds to 300 nm. (*b*) Frequency distribution regarding the positional shifts of the estimated center-of-symmetry in 1000 diffraction patterns from the positions of the direct-beam given initially in the simulation. (*c*) Comparison of the frequency distribution on the center-of-symmetry positions of 1000 times analyses for patterns β (green colored bars) and α (red) in (*a*). (*d*) Schematic illustration of the geometrical relationship between the direct-beam positions in the MPCCD-Octal and the MPCCD-Dual detectors. The details are described in the text.

Here we discuss whether equation (2) is applicable to various types of speckle patterns to determine the direct-beam positions in diffraction patterns through a set of simulations shown in Fig. 10(*a*) ▶. For 1000 noise-containing diffraction patterns calculated from assemblies of circular objects, the positions of the centers of symmetry which give the highest *C*_sym_ scores coincide well with the correct direct-beam positions within an ambiguity of 0.5 pixel at the MPCCD-Octal detector (Fig. 10*b* ▶). In diffraction pattern β in Fig. 10(*a*) ▶, the direct-beam position is somewhat difficult to determine because of the weak and the small number of speckles. However, the direct-beam positions are determined within an error range of 0.5 pixel (Fig. 10*c* ▶), although the rate of error is slightly larger than that for pattern α, the direct-beam position of which is easily determined owing to the clear and intense speckles. Thus, the algorithm may be applicable to determine the direct-beam positions of diffraction patterns pulse by pulse.

In near-future experiments at SACLA, we plan for the camera length of the MPCCD-Dual detector to be twice that currently in use, to extend the dynamic range of the detector system. Then, we would attempt a simple geometrical approximation between the direct-beam positions of the MPCCD-Octal and the MPCCD-Dual detectors (Fig. 10*d* ▶),
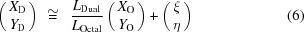
where 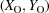 is the beam center position in the diffraction pattern recorded by the Octal detector, and 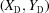 is that in the Dual detector. 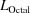 and 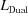 are the camera lengths of the Octal and the Dual detectors, respectively. Parameters 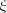 and 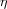 are constants for each experimental set-up.

This equation is advantageous in that it reduces the computation time by omitting the procedure to search the positions of center-of-symmetry in the diffraction patterns of the MPCCD-Dual detector. In addition, using this scheme, we can determine simply and rapidly the direct-beam position of the diffraction pattern recorded by the MPCCD-Dual detector, even when a number of pixels around the beamstop are in saturation.

### 6.2. Stability of the direct-beam position at beamline 3 of SACLA   

The stability of the direct-beam position has been reported roughly within the range of 19 µm in the vertical direction and 40 µm in the horizontal direction upstream of the experimental hutch (Tono *et al.*, 2013 ▶). In our experiments, for at least several hours, the direct-beam position estimated using equation (2) fluctuates within approximately 50 µm (Fig. 6*c* ▶), indicating the stability of the X-ray beam position at beamline 3 of SACLA.

Since speckle patterns of non-crystalline particles are broad in comparison with Bragg diffraction spots from crystals, further unambiguous determination of the direct-beam position using equation (2) is difficult. Collection of diffraction patterns from protein crystals is the best way to monitor the stability of the direct-beam position for a long period of time. The algorithm used in the indexing and integration of diffraction patterns sensitively determines the direct-beam positions (Rossmann, 1985 ▶). The stabilized direct-beam position is advantageous for collecting diffraction patterns at the very small angles necessary for phase-retrieval calculations.

### 6.3. Analysis of size distribution of sub-micrometer particles   

Using the *SITENNO* suite and KOTOBUKI-1 in XFEL-CXDI experiments, we are able to construct a size distribution of copper oxide particles as well as their internal structures from thousands of diffraction patterns in a short period of time (Fig. 9*b* ▶). In material science and industry, the physicochemical properties of nanoparticles are known to correlate with the internal structure and the size (Kottmann *et al.*, 2001 ▶). Thus, the size distribution of sub-micrometer particles is analyzed using various measurement techniques.

Dynamic light scattering (DLS), small-angle X-ray scattering (SAXS) and electron microscopy are popular for determining the size distribution of nanoparticles. The DLS technique provides the range of Stokes radii of particles through time-correlation analysis for light scattering from particles in Brownian motion in solution. For a monodispersive solution of particles, SAXS reports the radii of gyration, the second moment of electron density distribution in particles. While DLS and SAXS give averaged information of particle sizes in solution, transmission electron microscopy gives the outer shapes of individual particles at high resolution. However, the penetration depth of electrons caused by interactions with atoms makes it difficult to visualize the internal structures of particles with dimensions larger than 100 nm.

Studies of the size distribution through CXDI experiments provide both the shape and internal structures. Diffraction data will be collected within a short period of time and the *SITENNO* suite provides the detailed structural results within a few hours for several thousands of diffraction data (Table 1 ▶). Thus, XFEL-CXDI using the KOTOBUKI-1 apparatus and the *SITENNO* suite offers a technique to characterize simultaneously the size distribution and the internal structures of sub-micrometer particles (Takahashi *et al.*, 2013 ▶). The complementary use of the current and the conventional measurement techniques may provide more fruitful structural information to design and control the structure and function of nano-metal particles in material science and industry as well as biological samples targeted in the near future.

### 6.4. Future improvement toward fully automatic data processing   

Currently we extract diffraction patterns where the sum of the diffraction intensities in the small-angle region is larger than a threshold intensity, which is given by the user. Through the accumulation of diffraction data of various specimens, we would acquire quantitative data regarding the threshold values to automatically apply collected diffraction data depending predominantly on the atom species composing the specimens and the X-ray intensity.

In addition, toward fully automatic data processing, we are planning to introduce an algorithm to determine the resolution limit using the signal-to-noise ratio in resolution shells. The algorithm can automatically judge whether each diffraction pattern is worth being processed and analyzed using a given signal-to-noise ratio regarding the maximum of the diffraction intensity in the resolution shell. This approach may be suitable for treating anisotropic diffraction patterns including those from copper oxide particles. We will extract diffraction patterns with defining resolution using the signal-to-noise ratio rather than the sum of the diffraction intensity in ROIs covering a wide resolution range. To examine this idea, we have been collecting diffraction patterns from various types of non-crystalline particles in material science and biology.

With the combined use of the KOTOBUKI-1 diffraction apparatus and the *SITENNO* data processing software suite, the ratio between the phase-retrieved diffraction patterns against the total number of patterns is about 14.6%. In future utilization of XFELs for non-crystalline particles, this ratio should be improved. For this purpose, it will be necessary to develop new algorithms for use with diffraction patterns with weak intensity for merging and structure analyses.

From the point of view of constructing experimental apparatus and preparing specimens, it is better to place the specimen particles in the irradiation area such that the center of the particle coincides with the intensity peak of the focused X-ray pulses. The positional stability of the X-ray beam at beamline 3 of SACLA is advantageous when mechanically positioning specimen particles in the irradiation cross-sectional area of the focused X-rays. This increases the possibility of collecting good diffraction data suitable for structure analysis.

## Figures and Tables

**Table 1:** Performance of the subprograms

Subprogram	CPU time for processing 1000 diffraction patterns
Conversion to HDF5 files	295s
*TAMON*	289s
*JIKOKU*/*KOHMOKU*	983s
*ZOCHO*	501s / 960 cores in SACLA-HPC
Total	2068s

**Table 2:** Parameters approximating the diffraction pattern of a copper oxide particle in the MPCCD-Octal and Dual detectors

Parameters	MPCCD-Octal	MPCCD-Dual
*K* (photons)	13825.0 64.1	922.22 4.48
*A* (nm)		125.16 0.14
*B* (nm)		130.76 0.14
()	15.29 0.02	14.92 0.09
*X*c (pixel)	259.58 0.03	253.77 0.06
*Y*c (pixel)	253.95 0.03	256.79 0.05
